# Isolation and Characterization of Dromedary Camel Coronavirus UAE-HKU23 from Dromedaries of the Middle East: Minimal Serological Cross-Reactivity between MERS Coronavirus and Dromedary Camel Coronavirus UAE-HKU23

**DOI:** 10.3390/ijms17050691

**Published:** 2016-05-07

**Authors:** Patrick C. Y. Woo, Susanna K. P. Lau, Rachel Y. Y. Fan, Candy C. Y. Lau, Emily Y. M. Wong, Sunitha Joseph, Alan K. L. Tsang, Renate Wernery, Cyril C. Y. Yip, Chi-Ching Tsang, Ulrich Wernery, Kwok-Yung Yuen

**Affiliations:** 1 State Key Laboratory of Emerging Infectious Diseases, the University of Hong Kong, Pokfulam, Hong Kong; skplau@hku.hk (S.K.P.L.); kyyuen@hku.hk (K.-Y.Y.); 2 Department of Microbiology, the University of Hong Kong, Pokfulam, Hong Kong; rachelfyy2004@yahoo.com.hk (R.Y.Y.F.); candylaucy@gmail.com (C.C.Y.L.); emilyhk2811@gmail.com (E.Y.M.W.); alantsangmb@gmail.com (A.K.L.T.); cyrilyip@gmail.com (C.C.Y.Y.); microbioct@connect.hku.hk (C.-C.T.); 3 Research Centre of Infection and Immunology, the University of Hong Kong, Pokfulam, Hong Kong; 4 Carol Yu Centre for Infection, the University of Hong Kong, Pokfulam, Hong Kong; 5 Collaborative Innovation Center for Diagnosis and Treatment of Infectious Diseases, Zhejiang University, Hangzhou 310006, China; 6 Central Veterinary Research Laboratory, Dubai, UAE; sjoseph@cvrl.ae (S.J.); wernery@cvrl.ae (R.W.)

**Keywords:** coronavirus, dromedary camel, isolation, characterization

## Abstract

Recently, we reported the discovery of a dromedary camel coronavirus UAE-HKU23 (DcCoV UAE-HKU23) from dromedaries in the Middle East. In this study, DcCoV UAE-HKU23 was successfully isolated in two of the 14 dromedary fecal samples using HRT-18G cells, with cytopathic effects observed five days after inoculation. Northern blot analysis revealed at least seven distinct RNA species, corresponding to predicted subgenomic mRNAs and confirming the core sequence of transcription regulatory sequence motifs as 5′-UCUAAAC-3′ as we predicted previously. Antibodies against DcCoV UAE-HKU23 were detected in 58 (98.3%) and 59 (100%) of the 59 dromedary sera by immunofluorescence and neutralization antibody tests, respectively. There was significant correlation between the antibody titers determined by immunofluorescence and neutralization assays (Pearson coefficient = 0.525, *p* < 0.0001). Immunization of mice using recombinant N proteins of DcCoV UAE-HKU23 and Middle East respiratory syndrome coronavirus (MERS-CoV), respectively, and heat-inactivated DcCoV UAE-HKU23 showed minimal cross-antigenicity between DcCoV UAE-HKU23 and MERS-CoV by Western blot and neutralization antibody assays. Codon usage and genetic distance analysis of RdRp, S and N genes showed that the 14 strains of DcCoV UAE-HKU23 formed a distinct cluster, separated from those of other closely related members of *Betacoronavirus 1*, including alpaca CoV, confirming that DcCoV UAE-HKU23 is a novel member of *Betacoronavirus 1*.

## 1. Introduction

Coronaviruses (CoVs) are found in a wide variety of animals [1] in which they can cause respiratory, enteric, hepatic, and neurological diseases. Based on genotypic and serological characterization, CoVs were traditionally classified into three distinct groups [2,3,4]. Recently, the Coronavirus Study Group of the International Committee for Taxonomy of Viruses has replaced the traditional groups 1, 2, and 3 CoVs with three genera, *Alphacoronavirus*, *Betacoronavirus*, and *Gammacoronavirus*, respectively [5]. As a result of their unique mechanism of viral replication, CoVs have a high frequency of recombination [2,6]. Their tendency for recombination and high mutation rates may allow them to adapt to new hosts and ecological niches [7,8,9,10].

In 2003, the severe acute respiratory syndrome (SARS) epidemic, discovery of SARS-CoV, and identification of SARS-CoV-like viruses from Himalayan palm civets from wild live markets in China boosted interest in the discovery of novel CoVs in humans and animals [11,12,13,14,15,16]. A novel human CoV (HCoV) of the genus *Alphacoronavirus*, human CoV NL63 (HCoV-NL63), was reported in 2004 [17,18,19]. In 2005, we also described the discovery, complete genome sequence, and molecular epidemiology of another novel HCoV, human CoV HKU1 (HCoV-HKU1), in the genus *Betacoronavirus* [20,21,22]. As for animal CoVs, we and others have described the discovery of SARS-CoV-like viruses in Chinese horseshoe bats in Hong Kong and other horseshoe bats in other provinces of China [23,24]. Recently, the discovery of SARS-CoV-like viruses in Chinese horseshoe bats in Yunnan has further highlighted the importance for hunting the animal origin of human infections [25]. In addition, we have also discovered 21 other animal CoVs, which include two novel lineages in *Betacoronavirus* and a novel genus *Deltacoronavirus* [26,27,28,29,30,31,32,33,34,35,36]. From our studies it was shown that bats are the gene source for *Alphacoronavirus* and *Betacoronavirus* and birds are the gene source for *Gammacoronavirus* and *Deltacoronavirus* to fuel CoV evolution and dissemination [33].

In 2012, a novel CoV, named Middle East respiratory syndrome coronavirus (MERS-CoV), closely related to *Tylonycteris* bat CoV HKU4 (Ty-BatCoV HKU4) and *Pipistrellus* bat CoV HKU5 (Pi-BatCoV HKU5), has emerged as a cause of severe respiratory infections associated with high mortalities [37,38,39,40]. It has been shown that dromedaries in the Middle East possessed neutralizing antibodies against MERS-CoV [41,42]. Furthermore, MERS-CoV was also detected in the nasal swabs of dromedaries in Qatar, Saudi Arabia, Egypt, and the United Arab Emirates [43,44,45,46,47]. The recent emergence of MERS and the discovery of MERS-CoV in dromedaries have boosted interest in the search for other novel viruses in dromedaries [48,49]. In a recent molecular epidemiology study, we discovered a novel betacoronavirus, named dromedary camel CoV UAE-HKU23 (DcCoV UAE-HKU23), from fecal samples of dromedaries from Dubai [50]. In this study, we report the isolation of DcCoV UAE-HKU23 from the fecal sample of a dromedary and its characterization.

## 2. Results

### 2.1. Isolation of DcCoV UAE-HKU23

**Figure 1:**
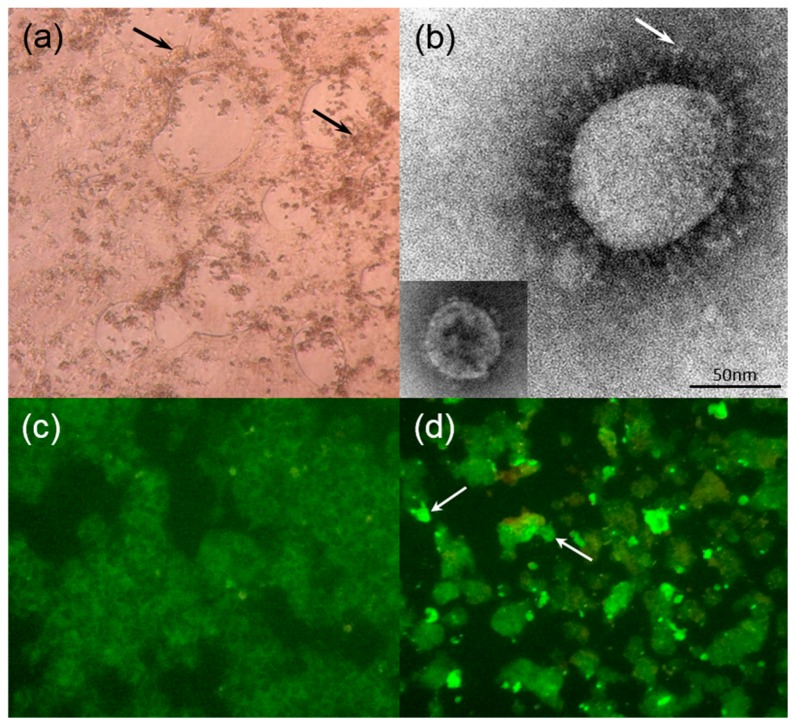
(**a**) HRT-18G cells infected with dromedary camel coronavirus (DcCoV) UAE-HKU23 showing cytopathic effects with rounded, aggregated, fused, and granulated giant cells rapidly detaching from the monolayer at day 5 after incubation (arrows) (original magnification 40×); (**b**) Negative contrast electron microscopy of ultracentrifuged deposit of HRT-18G cell culture-grown DcCoV UAE-HKU23, showing typical club-shaped surface projections (arrow) of coronavirus particles, with rabbit coronavirus HKU14 as the control (bottom **left** corner). Bar = 50 nm; Indirect immunofluorescent antigen detection in (**c**) uninfected and (**d**) infected HRT-18G cells using serum from dromedary showing apple green fluorescence in (arrows) DcCoV UAE-HKU23 infected HRT-18G cells (original magnification 100× for both).

Of the seven cell lines inoculated with dromedary fecal samples positive for DcCoV UAE-HKU23, viral replication was detected by RT-PCR in the supernatants of HRT-18G in two of the 14 dromedary fecal samples at day 7, with viral loads of 2.6 × 10^1^^0^ and 9.7 × 10^6^ copies/mL in HRT-18G cells in the presence of trypsin. Cytopathic effects (CPE), mainly in the form of rounded, fused and granulated giant cells rapidly detaching from the monolayer, were also observed in infected HRT-18G cells five days after inoculation (Figure 1a), which showed viral nucleocapsid expression by immunofluorescence in 20% of cells. Electron microscopy of ultracentrifuged cell culture extracts from infected HRT-18G cells showed the presence of CoV-like particles around 70–100 nm in diameter with typical club-shaped surface projections (Figure 1b).

### 2.2. Subgenomic mRNAs and Their Leader-Body Junction Sequences

**Figure 2:**
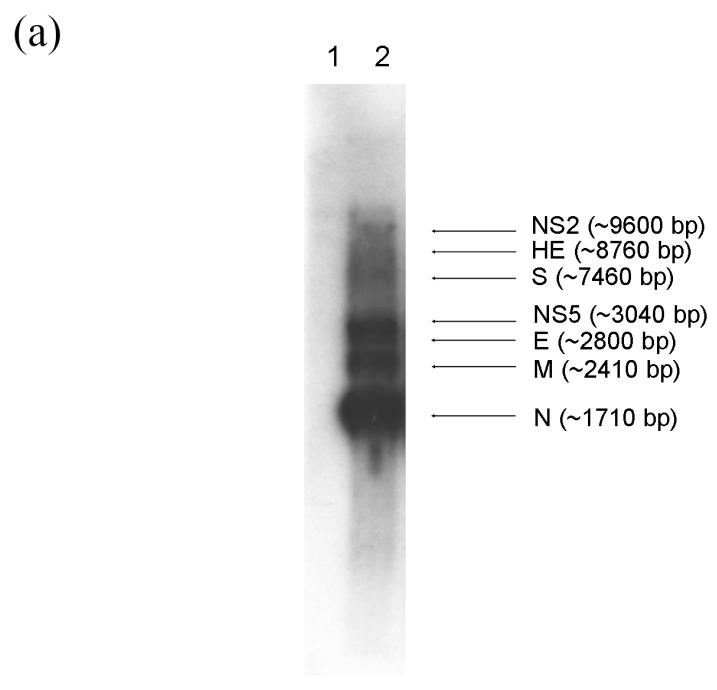

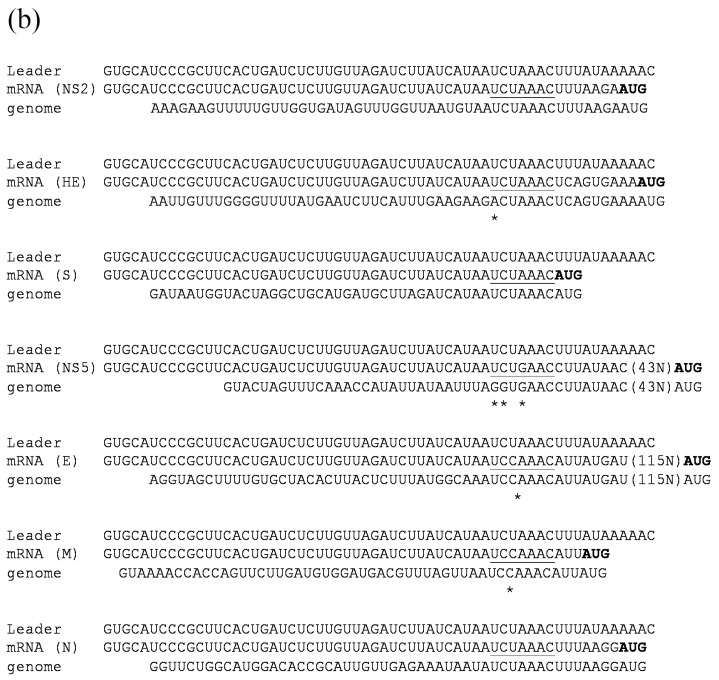
(**a**) Northern blot analysis for total RNA isolated from dromedary camel coronavirus (DcCoV) UAE-HKU23–infected HRT-18G cells. RNA species are indicated by arrows. NS2, non-structural NS2; HE, hemagglutinin; S, spike; NS5, non-structural NS5; E, envelope; M, membrane; N, nucleocapsid. Lane 1, 1 μg total RNA from uninfected cells; Lane 2, 1 μg total RNA from infected cells; (**b**) DcCoV UAE-HKU23 subgenomic mRNA (sg mRNA) leader-body junction and flanking sequences. The subgenomic mRNA sequences are shown in alignment with the leader and the genomic sequences. The start codon AUG in each subgenomic mRNA is depicted in bold. The putative transcription regulatory sequences (TRS) was underlined and base mismatch between the body TRS and the leader TRS or the corresponding genomic region was indicated by asterisk. The 43N and 115N in the parentheses indicate that 43 and 115 nucleotides at that region are not shown.

CoVs are characterized by a unique mechanism of discontinuous transcription with the synthesis of a nested set of subgenomic mRNAs [51,52]. To assess the number and size of DcCoV UAE-HKU23 subgenomic mRNA species, Northern blot analysis with a probe specific to the nucleocapsid sequence was performed. At least seven distinct RNA species were identified, with the sizes corresponding to predicted subgenomic mRNAs of NS2 (~9600 bp), hemagglutinin-esterase (HE) (~8760 bp), spike (S) (~7460 bp), NS5 (~3040 bp), envelope (E) (~2800 bp), membrane (M) (~2410 bp) and nucleocapsid (N) (~1710 bp) (Figure 2a).

By determining the leader-body junction sequences of subgenomic mRNAs from DcCoV UAE-HKU23–infected cell cultures, the subgenomic mRNA sequences were aligned to the leader sequence which confirmed the core sequence of the transcription regulatory sequences (TRS) motifs as 5′-UCUAAAC-3′ (Figure 2b), as in other *Betacoronavirus* lineage A CoVs [53,54,55,56]. The leader TRS and subgenomic mRNA of NS2, HE, S, and N exactly matched each other, whereas there was one base mismatch for NS5, E, and M. These results are in line with our previous prediction on the TRS [50]. The DcCoV UAE-HKU23 common leader on subgenomic mRNAs was confirmed as the first 65 nucleotides of the DcCoV UAE-HKU23 genome.

### 2.3. Immunofluorescence Antibody Tests and Neutralization Antibody Assays

**Figure 3:**
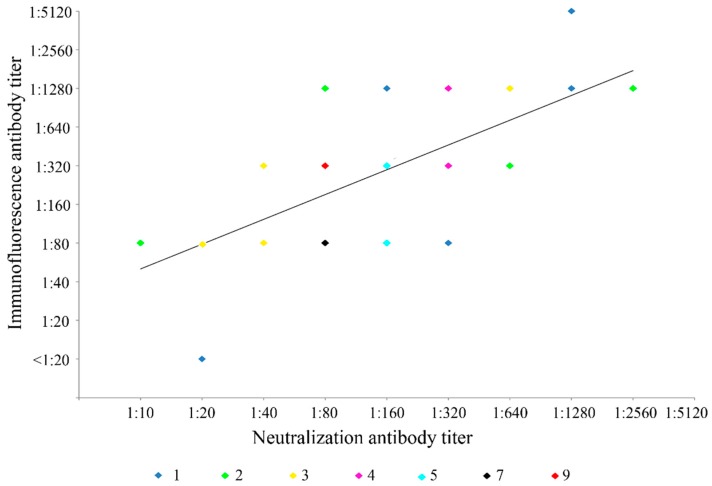
Comparison of neutralization antibody titer and immunofluorescence antibody titer of dromedary serum samples for dromedary camel coronavirus UAE-HKU23. Numbers of serum samples with that particular neutralization antibody and immunofluorescence antibody titers are indicated by points with different colors.

Fifty-nine serum samples from dromedaries were subject to immunofluorescence and neutralization antibody assays for DcCoV UAE-HKU23. Results were positive in 58 (98.3%) and 59 (100%) of the 59 samples as detected by immunofluorescence and the neutralization antibody test, respectively (Table 1; Figure 1c,d). In addition, there was significant correlation between the titers determined by the immunofluorescence antibody test and the neutralization antibody assay (Pearson coefficient = 0.525, *p* < 0.0001) (Figure 3).

### 2.4. Cross-Antigenicity between DcCoV UAE-HKU23 and MERS-CoV

**Figure 4:**
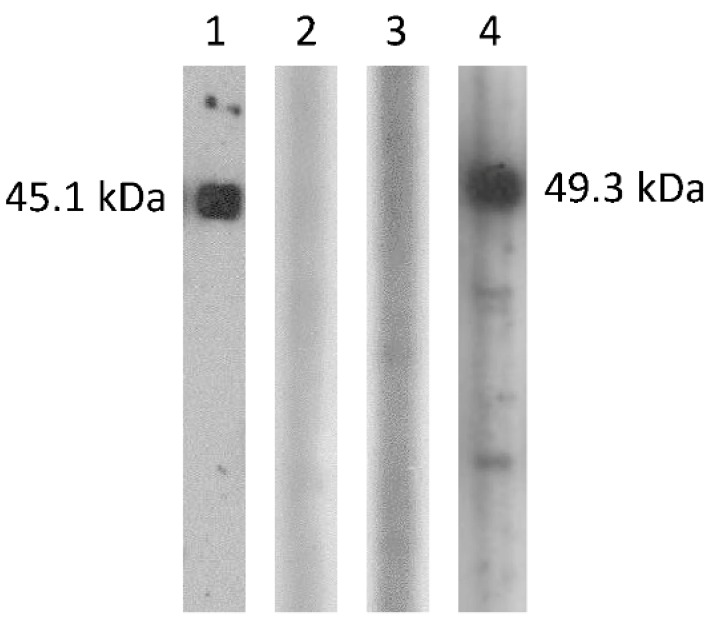
Western blotting analysis of dromedary camel coronavirus (DcCoV) UAE-HKU23 and Middle East respiratory syndrome coronavirus (MERS-CoV) N proteins expressed in *E. coli*. Lane 1: MERS-CoV N protein reacted with 1:16,000 dilution of serum from mouse immunized with MERS-CoV N protein; Lane 2: MERS-CoV N protein reacted with 1:16,000 dilution of serum from mouse immunized with DcCoV UAE-HKU23 N protein; Lane 3: DcCoV UAE-HKU23 N protein reacted with 1:16,000 dilution of serum from mouse immunized with MERS-CoV N protein, Lane 4: DcCoV UAE-HKU23 N protein reacted with 1:16,000 dilution of serum from mouse immunized with DcCoV UAE-HKU23 N protein.

To examine possible cross-antigenicity between the DcCoV UAE-HKU23 and MERS-CoV N proteins, recombinant N proteins of DcCoV UAE-HKU23 and MERS-CoV were cloned and purified and tested against serum samples of balb/c mice immunized with the N proteins of DcCoV UAE-HKU23 and MERS-CoV, respectively. The highest dilutions of the serum, obtained from the mouse immunized with the DcCoV UAE-HKU23 N protein, which generated immunoreactive bands in the Western blot assay, were 1:64,000 and 1:2000, respectively, when the DcCoV UAE-HKU23 N protein and MERS-CoV N protein were used as the antigens, respectively (Figure 4). The highest dilutions of the serum, obtained from the mouse immunized with the MERS-CoV N protein, which generated immunoreactive bands in the Western blot assay, were 1:512,000 and 1:8000, respectively, when the MERS-CoV N protein and DcCoV UAE-HKU23 N protein were used as the antigens, respectively (Figure 4).

To further examine possible cross-antigenicity between DcCoV UAE-HKU23 and MERS-CoV, heat-inactivated DcCoV UAE-HKU23 was used for immunization of balb/c mice and the serum obtained was tested for neutralization antibodies against DcCoV UAE-HKU23 and MERS-CoV, respectively. Neutralization antibody assays showed that the neutralization antibody titer of the pooled serum sample was 1:160 for DcCoV UAE-HKU23 but <1:20 for MERS-CoV.

### 2.5. Complete RdRp, S and N Gene Sequence Analysis

**Figure 5:**
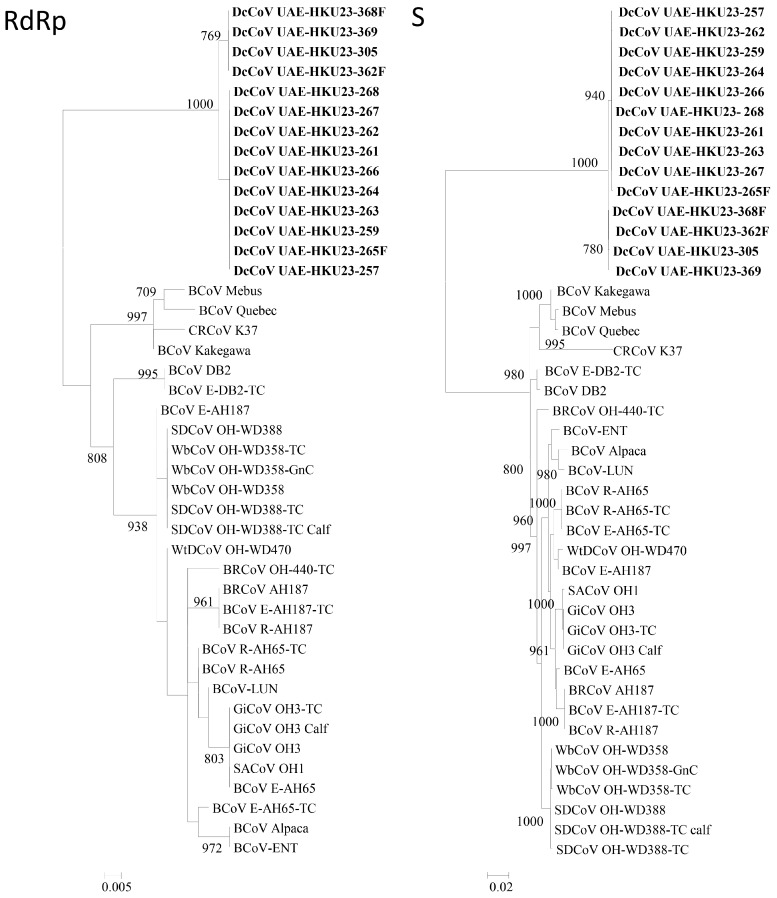

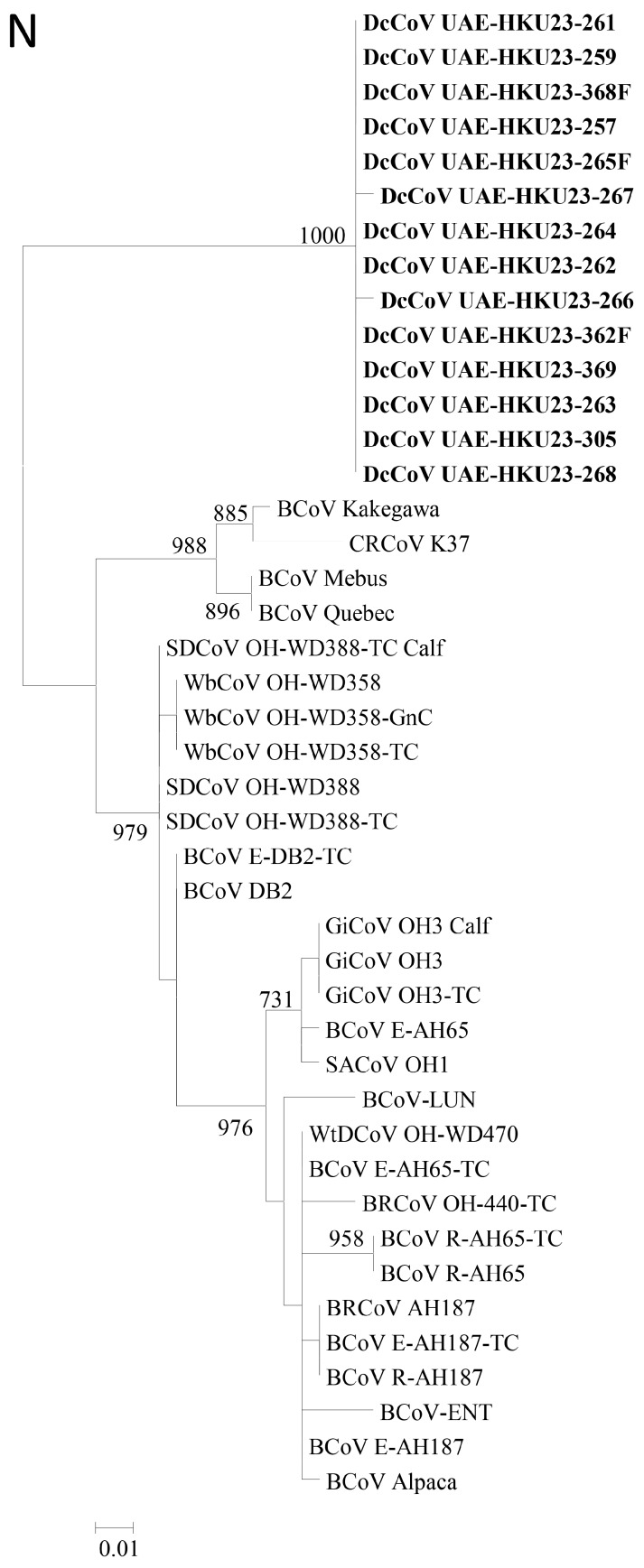
Phylogenetic analyses of RNA-dependent RNA polymerase (RdRp), S, and N genes of dromedary camel coronavirus (DcCoV) UAE-HKU23. Included in the analysis were 2784, 4101, and 1347 nucleotide positions in RdRp, S and N, respectively. For RdRp, the scale bar indicates the estimated number of substitutions per 200 nucleotides. For S, the scale bars indicate the estimated number of substitutions per 50 nucleotides. For N, the scale bars indicate the estimated number of substitutions per 100 nucleotides. Bootstrap values were calculated from 1000 trees and those below 70% are not shown. The 14 strains of DcCoV UAE-HKU23 characterized in this and our previous studies are shown in bold. BCoV, bovine coronavirus; CRCoV, canine respiratory coronavirus; SDCoV, sambar deer coronavirus; WbCoV, waterbuck coronavirus; WtDCoV, white-tailed deer coronavirus; BRCoV, bovine respiratory coronavirus; GiCoV, giraffe coronavirus; SACoV, sable antelope coronavirus.

To determine the number of genotypes/circulating strains of DcCoV UAE-HKU23, the complete RNA-dependent RNA polymerase (RdRp), S, and N genes of 11 additional DcCoV UAE-HKU23 strains from dromedary fecal samples positive for DcCoV UAE-HKU23 were sequenced. For the RdRp and N genes, all these 11 DcCoV UAE-HKU23 strains together with those of the three DcCoV UAE-HKU23 strains with complete genome sequences [50] possessed identical amino acid sequences, although there were 0–2 nucleotide differences among the strains for both genes (Figure 5). As for the S gene, phylogenetic analysis using either nucleotide or amino acid sequences revealed two clusters (Figure 5). There were 10 nucleotide differences among the two groups. Five were synonymous substitutions, and the other five resulted in amino acid changes (V105A, S436A, L845V, S967P, and P1188S).

### 2.6. Codon Usage Analysis and Genetic Distance of RdRp, S and N Genes

**Figure 6:**
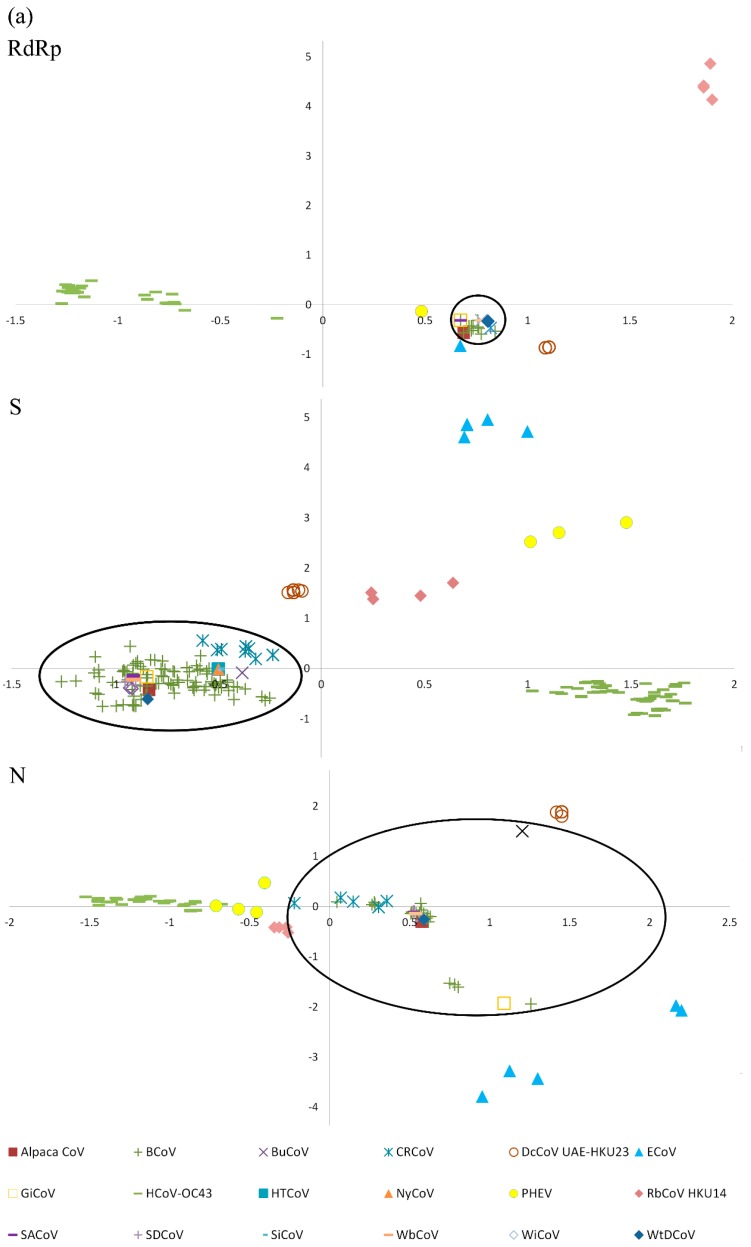

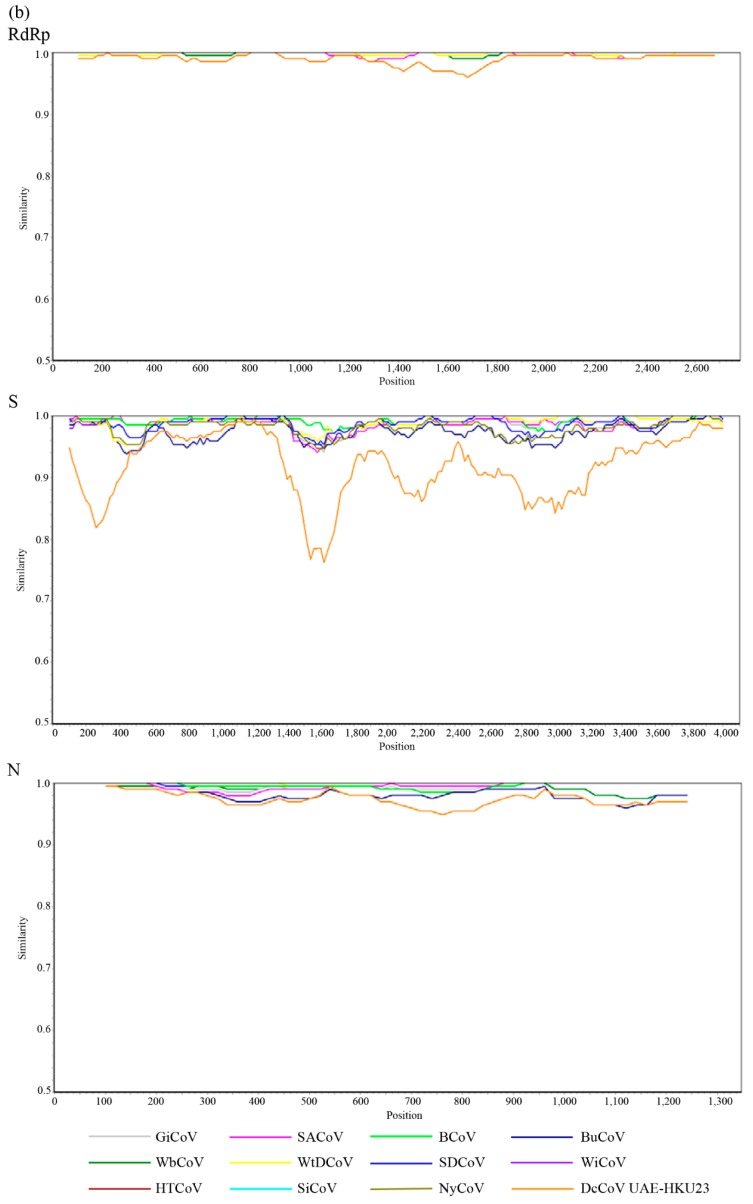
(**a**) Scatter plot of the corresponding analysis (CA) using relative synonymous codon usage (RSCU) of the RdRp, S, and N genes of members of *Betacoronavirus 1* and RbCoV HKU14. Different coronaviruses are indicated in different colored markers. The group of bovine coronavirus-like viruses is circled; (**b**) SimPlot analysis of complete RdRp, S, and N genes of DcCoV UAE-HKU23, alpaca CoV, BCoV and other wild ruminant CoVs. Each point plotted is the percent genetic distance within a sliding window of 200 nt wide, centered on the position plotted, with a step size of 20 nt. Each curve represents a comparison of the sequence data of DcCoV UAE-HKU23, BCoV, and other wild ruminant CoV strains to the reference sequence data of alpaca CoV. Alpaca CoV, alpaca coronavirus; BCoV, bovine coronavirus; BuCoV, *Bubalus bubalis* coronavirus; CRCoV, canine respiratory coronavirus; DcCoV, dromedary camel coronavirus, ECoV, equine coronavirus; GiCoV, giraffe coronavirus; HCoV-OC43, human coronavirus OC43; HTCoV, Himalayan tahr coronavirus; NyCoV, nyala coronavirus; PHEV, porcine hemagglutinating encephalomyelitis virus; RbCoV, rabbit coronavirus; SACoV, sable antelope coronavirus; SDCoV, sambar deer coronavirus; SiCoV, sitatunga coronavirus; WbCoV, waterbuck coronavirus; WiCoV, wisent coronavirus; WtDCoV, white-tailed deer coronavirus.

By corresponding analysis (CA) on relative synonymous codon usage (RSCU) values, patterns in codon usage were observed that allowed different groups in *Betacoronavirus 1* to be distinguished. The RdRp, S, and N genes of the 14 strains of DcCoV UAE-HKU23 formed a distinct cluster, separated from those of other closely related members of *Betacoronavirus 1*, including alpaca CoV (DQ915164) (Figure 6a) [57]. Analysis of the nucleotide sequence corresponding to the RdRp, S, and N genes showed that alpaca CoV has a lower genetic distance to bovine CoV (BCoV) and other recently isolated wild ruminant CoVs than to DcCoV UAE-HKU23 (Figure 6b).

## 3. Discussion

In this study, we have successfully isolated DcCoV UAE-HKU23 using the HRT-18G cell line. With only a few exceptions, such as SARS-CoV and MERS-CoV, CoVs are notoriously difficult to isolate in cell lines [58,59]. For the HCoVs OC43 and 229E, they induce only subtle CPEs, even if isolated. In our previous report on the discovery of DcCoV UAE-HKU23 from fecal samples of dromedaries by RT-PCR, no positive culture results were obtained from any of the three samples used for complete genome sequencing [50]. In the present study, after inoculation of all 14 fecal samples that were RT-PCR–positive for DcCoV UAE-HKU23, the virus was successfully isolated from two of the 14 samples using HRT-18G cells, with rounding and fusion to giant cells rapidly detaching from the monolayer. Northern blot experiments and determination of leader-body junction sequences of subgenomic mRNAs confirmed our previous predictions on the open reading frames (ORFs) and TRS of DcCoV UAE-HKU23 by comparison to other CoVs of *Betacoronavirus 1* [50]. Although sequencing of the RdRp, S, and N genes showed that there were two or more strains circulating in the dromedaries, no obvious genotypes were observed for the 14 strains of DcCoV UAE-HKU23 in this study. Since HCoV-OC43, BCoV [60], equine CoV, alpaca CoV, the murine hepatitis virus-H2 variant, our recently discovered rabbit CoV (RbCoV) HKU14 [34], and DcCoV UAE-HKU23 can all replicate in HRT-18G cells, it suggests that these CoVs that belonged to *Betacoronavirus* lineage A (the traditional “group 2 CoVs”) may share similar cellular tropisms. The HRT-18G cell line could be used to isolate other members of *Betacoronavirus* lineage A for further characterization of these CoVs.

Although DcCoV UAE-HKU23 and alpaca CoV are both isolated from camelids, they represent two distinct CoVs. Camelids (family *Camelidae*) are classified into the old world camels (genus *Camelus*), including dromedaries and Bactrians, and the new world camels (genera *Lama* and *Vicugna*), including llama, guanaco, alpaca, and vicuna. Since the alpaca CoV, which infects alpaca, and DcCoV UAE-HKU23, which infects dromedaries, are both members of *Betacoronavirus 1*, it raised the question whether alpaca CoV and DcCoV UAE-HKU23 are the same or different CoVs. We therefore analyzed the codon usage bias in the RdRp, S, and N genes of these two viruses. Results showed distinct codon usage bias and genetic distance for alpaca CoV and DcCoV UAE-HKU23. In fact, alpaca CoV showed much more similar codon usage bias and lower genetic distance to BCoV and other wild ruminant CoVs including water buffalo CoV, giraffe CoV, Himalayan tahr CoV, nyala CoV, sable antelope CoV, sambar deer CoV, sitatunga CoV, waterbuck CoV, wisent CoV, and white-tailed deer CoV than to DcCoV UAE-HKU23 in the RdRp, S, and N genes (Figure 6) [10,61,62,63,64]. In addition to codon usage and genetic distance, the S protein of DcCoV UAE-HKU23 contains one additional potential *N*-glycosylation site at amino acid position 492 compared to alpaca CoV (data not shown). This potential *N*-glycosylation site is also present in the S proteins of canine respiratory coronavirus and HCoV-OC43, but not in those of other wild ruminant CoVs. All these indicated that alpaca CoV and DcCoV UAE-HKU23 are two different CoVs adapting to two different hosts residing in two different geographical locations (alpaca in South America and dromedaries in the Middle East and North Africa) in the world.

Minimal cross-antigenicity was observed between the N proteins of DcCoV UAE-HKU23 and MERS-CoV. In our study on genotyping and serological characterization of *Rousettus* bat CoV HKU9 (Ro-BatCoV HKU9), we have shown that there is minimal serological cross-reactivity among the four lineages of betacoronaviruses using human serum samples positive for antibodies against the N protein of HCoV-HKU1 (lineage A), Chinese horseshoe bat serum samples positive for antibodies against the N protein of SARSr-Rh-BatCoV (lineage B), and Leschenault’s rousette bat serum samples positive for antibodies against the N protein of Ro-BatCoV HKU9 (lineage D), and against recombinant N proteins of HCoV-HKU1 (lineage A), SARSr-Rh-BatCoV (lineage B), Ty-BatCoV HKU4 (lineage C), Pi-BatCoV HKU5 (lineage C), and Ro-BatCoV HKU9 (lineage D) [31]. In our recent study on the discovery of DcCoV UAE-HKU23, the serological data further showed little cross-reactivity between DcCoV UAE-HKU23 (lineage A) and SARSr-Rh-BatCoV (lineage B), Pi-BatCoV HKU5 (lineage C), and Ro-BatCoV HKU9 (lineage D), respectively, using dromedary serum samples positive for antibodies against the N protein of DcCoV UAE-HKU23 [50]. In the present study, we showed that the antibody titer of the serum obtained from the mouse immunized with the recombinant N protein of MERS-CoV for the anti-N of MERS-CoV was 64 folds higher than that for the anti-N of DcCoV UAE-HKU23. Similarly, the antibody titer of the serum obtained from the mouse immunized with the recombinant N protein of DcCoV UAE-HKU23 for the anti-N of DcCoV UAE-HKU23 was 32 folds higher than that for the anti-N of MERS-CoV. Furthermore, for the mice immunized with heat-inactivated DcCoV UAE-HKU23, the neutralization antibody titer against DcCoV UAE-HKU23 was at least 16 folds higher than that against MERS-CoV. All these results confirmed that there is minimal serological cross-reactivity among CoVs of different lineages in *Betacoronavirus*, supporting their classification as separate lineages under *Betacoronavirus*.

A high seroprevalence for DcCoV UAE-HKU23 was observed in dromedaries. Since dromedaries are the natural reservoirs of both DcCoV UAE-HKU23 and MERS-CoV, the seroprevalence of these CoVs can only be ascertained if there is minimal serological cross-reactivity between these two viruses. Based on the minimal serological cross-reactivity between DcCoV UAE-HKU23 and MERS-CoV shown in the present study, we confirmed a high seroprevalence for DcCoV UAE-HKU23 in dromedaries as determined by immunofluorescence antibody tests and neutralization antibody assays. The relatively lower seropositivity as determined by Western blot in our previous study as compared to the very high seropositivity as determined by immunofluorescence antibody tests and neutralization antibody assays in the present study is because only prominent immunoreactive bands were considered unambiguously positive in Western blot analysis and hence might have underestimated the true seroprevalence [50].

## 4. Materials and Methods

### 4.1. Virus Culture

Original fecal samples from all the 14 dromedaries tested positive for DcCoV UAE-HKU23 by RT-PCR [50] were subject to virus isolation in HRT-18G (human rectal tumor epithelial), RK13 (rabbit kidney), MDCK (canine kidney), MDBK (bovine kidney), Dubca (Dubai camel fetus skin fibroblast), Caki-3-R (camel kidney), and BSC-1 (African green monkey renal epithelial) cells as described previously [65]. Cell lines were prepared in culture plates and inoculated with 200 μL of fecal samples diluted at 1:10. Non-attached viruses were removed by washing the cells twice in phosphate-buffered saline. The monolayer cells were maintained in serum-free minimal essential medium (MEM, Invitrogen, Carlsbad, CA, USA) with or without supplementation by tosylsulfonyl phenylalanyl chloromethyl ketone (TPCK)-treated trypsin (1 μg/mL) (Sigma, St. Louis, MO, USA). All infected cell lines were incubated at 37 °C for seven days. CPEs were examined at day 1, 3, 5, and 7 by inverted light microscopy.

### 4.2. Real-Time Quantitative RT-PCR

The assay was performed using a real-time one-step qRT-PCR with DcCoV UAE-HKU23 primers 5′-ATAGCGGCTACACGTGGTGTT-3′ and 5′-TCCCAGCCGCCATAAAACT-3′ and probe 5′-(FAM) CTGTTGTTATAGGCACCACT (BHQ1)-3′. To generate calibration curves, we prepared a series of six log_10_ dilutions equivalent to 10^1^–10^6^ copies per reaction mixture and ran them in parallel with the test samples.

### 4.3. Electron Microscopy

Negative contrast electron microscopy was performed as described previously [66,67]. Tissue culture cell extracts infected with DcCoV UAE-HKU23 were centrifuged at 19,000× *g* at 4 °C, after which the pellet was resuspended in phosphate-buffered saline and stained with 2% phosphotungstic acid. Samples were examined with a Philips EM208s electron microscope (Royal Philips, Amsterdam, The Netherlands).

### 4.4. Northern Blotting

Total RNA was extracted from DcCoV UAE-HKU23-infected HRT-18G cells using TRIzol Reagent (Invitrogen, Carlsbad, CA, USA). RNA was separated on 1% agarose gel with 7% formaldehyde at 100 V in 1× 3-(*N*-morpholino)propanesulfonic acid (MOPS) buffer (20 mM MOPS, 5 mM sodium acetate, 1 mM EDTA), transferred to a positively charged nylon membrane (Roche Diagnostics, Basel, Switzerland) with Transfer Buffer (Ambion, Foster City, CA, USA) by means of capillary force for 2 h, cross-linked to the membrane by ultraviolet light (120 mJ/cm^2^) and baked at 80 °C for 1 h. The blot was prehybridized with ULTRAhyb-Oligo Hybridization buffer (Ambion, USA) and probed with a DcCoV UAE-HKU23 nucleocapsid-specific oligodeoxynucleotide probe 5′-CCAGAACGATTTCCAGAGGACGCTCTACT-3′, which was labeled with digoxigenin (DIG) at 3′ end. The blot was hybridized at 42 °C overnight and washed with low- and high-stringency buffers as recommended by the manufacturer (Ambion). Detection of DIG-labeled probe on the blot was performed using DIG Luminescent Detection Kit according to manufacturer’s protocol (Roche Diagnostics).

### 4.5. Determination of Leader-Body Junction Sequence

To determine the location of the leader and body TRSs used for DcCoV UAE-HKU23 mRNA synthesis, the leader-body junction sites and flanking sequences of all DcCoV UAE-HKU23 subgenomic mRNAs were determined using RT-PCR as described previously [56,68]. Briefly, intracellular RNA was extracted from DcCoV UAE-HKU23-infected HRT-18G cells using TRIzol Reagent (Invitrogen). Reverse transcription was performed using random hexamers and the SuperScript III kit (Invitrogen). cDNA was PCR amplified with a forward primer located in the leader sequence and a reverse primer located in the body of each mRNA (Table 2). The PCR mixture (25 μL) contained cDNA, PCR buffer (10 mM Tris-HCl pH 8.3, 50 mM KCl, 2 mM MgCl_2_, and 0.01% gelatin), 200 μM of each dNTPs, and 1.0 U Taq polymerase (Applied Biosystems, Foster City, CA, USA). The mixtures were amplified in 40 cycles of 94 °C for 1 min, 50 °C for 1 min, and 72 °C for 1 min, and a final extension at 72 °C for 10 min in an automated thermal cycler (Applied Biosystems). RT-PCR products corresponding to each subgenomic mRNA could be distinguished by size differences on agarose gel electrophoresis. PCR products were gel-purified using QIAquick gel extraction kit (QIAGEN, Hilden, Germany) and sequenced to obtain the leader-body junction sequences for each subgenomic mRNA.

### 4.6. Immunofluorescence Antibody Tests

HRT-18G cells infected with DcCoV UAE-HKU23 were fixed in chilled acetone at −20 °C for 10 min. The fixed cells were incubated with four-fold dilutions of serum from 1:10 to 1:10,240 from 59 dromedaries, followed by 1:50 diluted Novex FITC-goat anti-llama IgG (Invitrogen). Cells were then examined under a fluorescence microscope. Uninfected cells were used as negative control.

### 4.7. Neutralization Antibody Assays

Neutralization antibody assays for DcCoV UAE-HKU23 were carried out according to previously described protocols with modifications [11,23]. Heat inactivated serum samples from dromedaries were serially diluted from 1:10 and then mixed with 100 50% tissue culture infective dose (TCID_50_) of DcCoV UAE-HKU23 isolate 263F. Available sera from 59 dromedaries were included. After incubation for 2 h at 37 °C, the mixture was inoculated in duplicate on to 96-well plates of HRT-18G cell cultures. Results were recorded after six days incubation at 37 °C.

### 4.8. Cloning and Purification of (His)_6_-Tagged Recombinant DcCoV UAE-HKU23 and MERS-CoV Nucleocapsid Proteins from Escherichia coli

To produce two plasmids for DcCoV UAE-HKU23 and MERS-CoV N proteins purification, primers (5′-CATGCCATGGGCATGTCTTTTACTCCTGGTAAGC-3′ and 5′-CCGCTCGAGTATTTCTGAGGTGTTTTCAG-3′ for DcCoV UAE-HKU23 and 5′-GGAATTCCATATGATGGCATCCCCTGCTGCACCTC-3′ and 5′-ATAAGAATGCGGCCGCATCAGTGTTAACATCAATCATT-3′ for MERS-CoV) were used to amplify the genes encoding the N genes of DcCoV UAE-HKU23 and MERS-CoV, respectively. The sequences encoding amino acids 1–449 and 1–414 of the N proteins of DcCoV UAE-HKU23 and MERS-CoV respectively were amplified and cloned into the NcoI and XhoI sites of expression vector pET-28b(+) and NdeI and NotI sites of expression vector pETH, respectively (Novagen, Madison, WI, USA). The recombinant N proteins of DcCoV UAE-HKU23 and MERS-CoV were expressed and purified using Ni^2+^-loaded HiTrap Chelating System (GE Healthcare, Buckinghamshire, UK) according to the manufacturer’s instructions.

### 4.9. Animal Immunization Using N Proteins of DcCoV UAE-HKU23 and MERS-CoV

To prepare antibodies specific against the N proteins of DcCoV UAE-HKU23 and MERS-CoV respectively, 30 μg of purified (His)_6_-tagged recombinant N proteins of DcCoV UAE-HKU23 or MERS-CoV was mixed with an equal part of complete Freund’s adjuvant and injected subcutaneously into balb/c (H-2^d^) mice (six to eight weeks old, 18 to 22 g). Incomplete Freund’s adjuvant was used in subsequent injections. Serum samples were taken two weeks after the third injection for Western blot analysis.

### 4.10. Western Blot Analysis

Western blot analysis was performed according to our published protocol [15], with 1.5 μg purified (His)_6_-tagged recombinant N proteins of DcCoV UAE-HKU23 and MERS-CoV, respectively. Serum samples of mice were titrated with serial two-fold dilutions, beginning at 1:2000. Antigen-antibody interaction was detected with 1:4000 diluted horseradish peroxidase-conjugated goat anti-mouse IgG (Novex, San Diego, CA, USA) and ECL fluorescence system (GE Healthcare, Buckinghamshire, UK).

### 4.11. Animal Immunization Using Heat-Inactivated DcCoV UAE-HKU23

DcCoV UAE-HKU23 was cultured in HRT-18G cells using serum-free MEM for four days and was heat-inactivated at 60 °C for 1 h. The cells were frozen and thawed once. The cell debris was removed by centrifugation and the supernatant was titrated and six balb/c mice were inoculated intraperitoneally using 1 × 10^5^ TCID_50_ of heat-inactivated DcCoV UAE-HKU23. Three boosters were administered on days 10, 20, and 30, respectively, with the heat inactivation of DcCoV UAE-HKU23 carried out on the day of injection. Serum samples were obtained 14 days after the fourth injection. The sera from the six mice were pooled and neutralization antibody assay for DcCoV UAE-HKU23 was performed as described above and that for MERS-CoV was performed as we described previously [69].

### 4.12. Sequencing of Complete RdRp, S, and N Genes of DcCoV UAE-HKU23 Strains

Viral RNA was extracted from the fecal samples using EZ1 Virus Mini Kit v2.0 (QIAGEN). The RNA was eluted in 60 μL of AVE buffer (QIAGEN) and was used as the template for RT-PCR.

The complete RdRp, S, and N genes of DcCoV UAE-HKU23 strains from 11 additional dromedaries were amplified and sequenced using primers designed by multiple alignments of the three complete genome sequences of DcCoV UAE-HKU23 [50]. Reverse transcription was performed using the SuperScript III kit. The PCR mixture (25 μL) contained DNA, iProof High-fidelity PCR buffer (Bio-Rad, Hercules, CA, USA), 200 μM of each dNTP, and 0.5 U iProof High-fidelity DNA polymerase (Bio-Rad). The mixtures were amplified by using 40 cycles of 98 °C for 10 s, 55 °C for 30 s, and 72 °C for 50 s, with a final extension at 72 °C for 10 min. Standard precautions were taken to avoid PCR contamination and no false-positive was observed in negative controls.

The PCR products were gel-purified using the QIAquick gel extraction kit (QIAGEN). Both strands of the PCR products were sequenced twice with an ABI Prism 3700 DNA Analyzer (Applied Biosystems), using the PCR primers. The sequences of the PCR products were assembled manually.

### 4.13. Phylogenetic Analysis

The phylogenetic trees for RdRp, S, and N genes were constructed by the maximum likelihood method using PhyML. The optimal model for the nucleotide sequences were determined using ModelGenerator v85. The trees for RdRp and N genes were inferred by the GTR + I substitution model and the tree for *S* gene was inferred by the GTR + I + G model.

### 4.14. Codon Usage Analysis and Genetic Distance of RdRp, S, and N Genes

The codon usage bias of the RdRp, S, and N genes of the members in *Betacoronavirus 1* and RbCoV HKU14 was estimated using RSCU. RSCU values were calculated for the 59 relevant codons (excluding methionine, tryptophan, and stop codons) by SSE [70]. CA was performed on the RSCU values using the R statistical software, version 2.15.1 [71] and the function “corresp” from the Modern Applied Statistics with S (MASS) library. The SimPlot program (version 3.5.1) was used to analyze the genetic distance of the RdRp, S, and N genes of DcCoV UAE-HKU23 and other wild ruminant CoVs in reference to the RdRp, S, and N genes of alpaca CoV, and this genetic distance was plotted *versus* nucleotide positions. The similarity plot was configured with window size: 200 nucleotides; step: 20; F84 (maximum likelihood).

## 5. Conclusions

DcCoV UAE-HKU23 was successfully isolated in dromedary fecal samples using HRT-18G cells. There was minimal serological cross-antigenicity between DcCoV UAE-HKU23 and MERS-CoV. DcCoV UAE-HKU23 is a novel member of *Betacoronavirus 1*.

## Figures and Tables

**Table 1:** Dromedary camel coronavirus UAE-HKU23 antibody detection by immunofluorescence and neutralization antibody tests.

Antibody Titer	Number (%) of Samples
Immunofluorescence Antibody Test	
<20	1 (1.7)
80	21 (35.6)
320	23 (39.0)
1280	13 (22.0)
5120	1 (1.7)
**Neutralization Antibody Test**	
10	2 (3.4)
20	4 (6.8)
40	6 (10.2)
80	18 (30.5)
160	11 (18.6)
320	9 (15.3)
640	5 (8.5)
1280	2 (3.4)
2560	2 (3.4)

**Table 2:** Primers used for RT-PCR amplification of the leader-body junction of subgenomic mRNAs (sg mRNAs) of dromedary camel coronavirus UAE-HKU23.

Primer	Sequence (5′-3′)	Use
LPW25800	GATTGTGAGCGATTTGCGTGC	Forward primer for all sg mRNAs PCR
LPW18463	GTAAACCTTTATAATTTAACACA	Reverse primer for mRNA (NS2) PCR
LPW25801	AATCGGTAAAGTGAAAACTCC	Reverse primer for mRNA (HE) PCR
LPW25802	CCACAATGTACTCAACAATAAAG	Reverse primer for mRNA (S) PCR
LPW25803	TAGCGAATGCTGTAAAACCAG	Reverse primer for mRNA (NS5) PCR
LPW25804	CTCATAAAACTGCCTACCTCT	Reverse primer for mRNA (E) PCR
LPW18468	CCAAGATACACATTATTCAACG	Reverse primer for mRNA (M) PCR
LPW18469	GAGTAATTCCAGAGAACCAAGA	Reverse primer for mRNA (N) PCR
